# Frontal Fibrosing Alopecia: A Histopathological Comparison of the Frontal Hairline with Normal-Appearing Scalp

**DOI:** 10.3390/jcm11144121

**Published:** 2022-07-15

**Authors:** María Librada Porriño-Bustamante, Fernando Javier Pinedo-Moraleda, Ángel Fernández-Flores, Trinidad Montero-Vílchez, María Antonia Fernández-Pugnaire, Salvador Arias-Santiago

**Affiliations:** 1 Dermatology Department, University Hospital La Zarzuela, 28023 Madrid, Spain; 2 Dermatology Department, University of Granada, 18016 Granada, Spain; 3 Pathology Department, University Hospital Alcorcón Foundation, 28922 Alcorcón, Spain; fernandojavier.pinedo@salud.madrid.org; 4 Pathology Department, University Hospital El Bierzo, 24404 Ponferrada, Spain; dermatopathonline@gmail.com; 5 Dermatology Department, University Hospital Virgen de las Nieves, 18014 Granada, Spain; tmonterov@gmail.com (T.M.-V.); salvadorarias@hotmail.es (S.A.-S.); 6 Dermatology Department, University Hospital San Cecilio, 18016 Granada, Spain; marian.fer@telefonica.net; 7 School of Medicine, Institute of Biosanitary Investigation (IBS), Granada University, 18016 Granada, Spain

**Keywords:** frontal fibrosing alopecia, scarring alopecia, histopathology

## Abstract

Frontal fibrosing alopecia is characterized by the presence of a lymphocytic inflammatory infiltrate around the upper follicle and by perifollicular fibrosis, which results in the destruction of the hair follicle. Recent reports have also found the presence of those findings in clinically unaffected areas. The aim of this report is to perform a deeper analysis of the histopathological features of this apparently unaffected scalp. A cross-sectional study including 52 women with frontal fibrosing alopecia was performed. Two areas were biopsied: the frontal hairline and a normal-appearing scalp area. Sebaceous glands were reduced/absent in 80.8% of the frontal hairline samples compared to 42.3% of the “healthy scalp” samples (*p* = 0.001). Inflammatory infiltrate was observed in 92.3% of patients in the frontal hairline and in 86.5% of them in the “healthy scalp” area (*p* = 0.508), although the severity was higher in the former (*p* = 0.013). Follicular epithelium changes were seen in 70.6% of the frontal hairline biopsies compared to 48.1% of the “healthy scalp” biopsies (*p* = 0.012). Fibrous tissular changes were noted in 80.8% and 53.8% of the frontal hairline and “healthy scalp” biopsies, respectively (*p* = 0.003). In conclusion, the histopathological features of frontal fibrosing alopecia are shared by both affected and clinically unaffected areas.

## 1. Introduction

Frontal fibrosing alopecia (FFA) is a lymphocytic scarring alopecia which has become the most common type of cicatricial alopecia [1]. Clinically, it is characterized by progressive frontal or temporoparietal, or even occipital, hairline recession, leading to an alopecic cicatricial band [2,3].

The loss of the immune privilege of the hair follicle is considered the starting point in the development of cicatricial alopecias [4,5]. After the exposure of the hair follicle to immune attacks, a cytotoxic T cell autoimmune reaction—induced by an unknown trigger—against the infundibular and isthmic regions, could lead to the damage of the stem cells of the bulge area and then to the irreversible destruction of the hair follicle. Histopathological typical features of FFA include a lichenoid lymphocytic infiltrate around the upper follicle, that is isthmus and infundibulum, as well as concentric perifollicular lamellar fibrosis [6,7]. Deep follicular involvement is less common but possibly to be found in FFA samples, and the interfollicular epidermis is usually spared [7,8,9].

Dermatologists can assess by the “naked-eye” and trichoscopy the “clinically affected area”, evaluating the size of the scarring area and the presence or absence of inflammatory trichoscopic signs. However, sonographic and histopathological abnormalities have been found in “normal-appearing” areas in FFA patients, suggesting that subclinical inflammation may exist [10,11,12,13,14]. The presence of the inflammatory infiltrate, sebaceous gland atrophy, and perifollicular fibrosis were the histopathological features found in the normal-appearing scalp; all of them are typical features found in the frontal hairline area in patients with FFA. The existence of histopathological abnormalities in areas of the scalp without clinical and trichoscopic inflammatory signs suggests that FFA may affect the entire scalp, although due to an unknown reason, only some areas develop the scarring alopecia. The published studies which compare the histopathological features in the frontal hairline and the “normal-appearing” scalp in FFA only assessed the presence and degree of the inflammatory infiltrate, the presence of mucin deposits, the atrophy of sebaceous glands, and the existence of perifollicular fibrosis.

The aim of this study is to make a broad comparison of the histopathological features of both clinically affected and “normal-appearing” areas in FFA. Up to now, this is the largest study of its kind. Moreover, the relation between the presence of the histopathological alterations in both areas with the existence of some specific clinical or trichoscopic signs has also been assessed.

## 2. Materials and Methods

A cross-sectional study was performed in the University Hospital San Cecilio in Granada (Spain). Inclusion criteria were as follows: women, with clinical and trichoscopic signs of FFA, that is, hairline recession which leads to a scarring band without follicular openings, with or without perifollicular erythema and follicular hyperkeratosis. Exclusion criterion was the male sex. All patients signed an informed consent and the study was approved by the Local Ethical Committee.

Demographic and clinical information were recorded, including age, age of onset of the alopecia, age of menopause, grade of the alopecia—following the five grades classification described by Vañó et al. [2]—, the duration of the alopecia, the presence of symptoms—pruritus, trichodynia—, the presence of facial papules and peripheral alopecia. Current treatment with corticosteroids was also registered. Moreover, the presence of trichoscopic signs, such as perifollicular erythema and follicular hyperkeratosis, was also recorded. A handheld dermoscope DermLite II pro HR was used.

Two dermoscopy-guided 4 mm punch scalp biopsies were performed on all patients. The first one was done in the frontal hairline (B1), in the most clinically affected area. The second one was done in a “normal-appearing” parietal area (B2), based on the absence of trichoscopic signs of FFA.

All specimens were divided and analyzed between two dermatopathologists, and then processed and stained with hematoxylin-eosin and cut in vertical sections. Orcein stain was used to assess elastic fibers.

The intensity of the inflammatory infiltrate was evaluated at a low power first, and then, attention to the specific location of it, was paid. Focal small patches of infiltrate were considered mild. Evidence of confluent patches of inflammatory infiltrate that did not cover in total more than 30% of the dermis, were considered as moderate. More extensive infiltrate was considered as severe.

Software SPSS (SPSS 25.0, SPSS Inc., Chicago, IL, USA) was used for data analyses. Student’s *t*-test was applied to compare mean values of quantitative variables. Qualitative histologic variables between both areas were analyzed with McNemar test. The other qualitative variables were analyzed with χ^2^ test, or the two-tailed Fisher exact test with 2 × 2 contingency tables in case of small samples. Differences were considered significant at *p* ≤ 0.05.

## 3. Results

A total of 52 women with FFA were included, which implied 104 scalp biopsies. The mean distance between both areas was 4 cm (1.4–17.6 cm, SD 3.26).

General characteristics of the patients are described in Table 1. With regards to current treatment for FFA, 26.9% (14/52) were not taking any, and only 36.5% (19/52) were using topical corticosteroids at the moment of the evaluation (twice a week) and 3.8% (2/52) were receiving intralesional corticosteroids injections every 3 months in addition to the topical therapy.

The hair count in the two scalp areas is described in Table 2. No differences were found regarding the presence of anagen, catagen, or telogen hairs in B1 compared to B2. Regarding the involvement of other skin annexes, rather than hair follicles (sebaceous glands and erector pili muscle), it was found in 86.5% (45/52) of B1 biopsies compared to 50% (26/52) of B2 biopsies (*p* < 0.001). In B1, sebaceous glands were normal in 19.2% (10/52), reduced in 30.8% (16/52), and absent in 50% (26/52), whereas in B2, normal sebaceous glands were observed in 57.7% (30/52), reduced in 15.4% (8/52), and absent in 26.9% (14/52) (*p* = 0.001). In relation to the erector pili muscle, in B1 it was normal in 76.9% (40/52) and reduced in 23.1% (12/52), while in B2 it was normal in 86.5% (45/52), reduced in 11.5% (6/52), and absent in 1.9% (1/52) (*p* = 0.227). Spared hair follicles were noted in 75% (39/52) of B1 samples compared to 92.3% (48/52) of B2 biopsies (*p* = 0.035).

**Figure 1:**
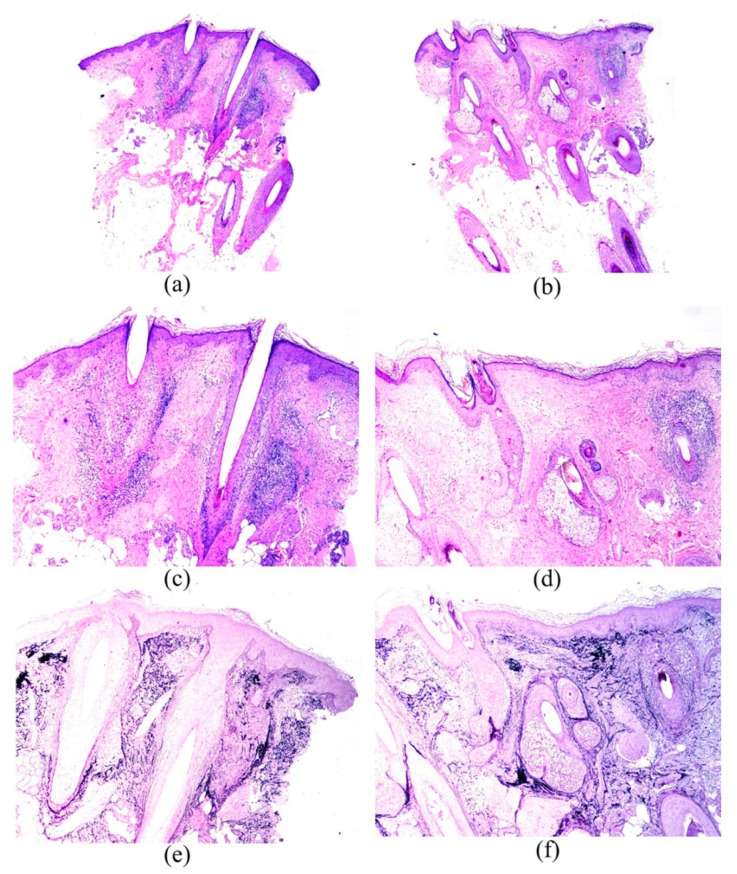
Images from patient number 50. (**a**,**c**,**e**) show the biopsy from the most clinically involved area (frontal hairline). Lymphoid infiltrate is prominent and erosion of the basal layer of the follicular istmus is remarkable. Orcein stain (**e**) shows the scarring in peri-infundibular areas. ((**a**) Hematoxylin-eosin × 20; (**c**) Hematoxylin-eosin × 40; (**e**) Shikatta orcein × 40)). (**b**,**d**,**f**) show the biopsies from a normal-appearing scalp area. The lymphoid infiltatrate is less prominent than in (**a**) and (**c**), and the scarring areas in (**f**) are less extensive than in (**e**). ((**b**) Hematoxylin-eosin × 20; (**d**) Hematoxylin-eosin × 40; (**f**) Shikatta orcein × 40).

**Figure 2:**
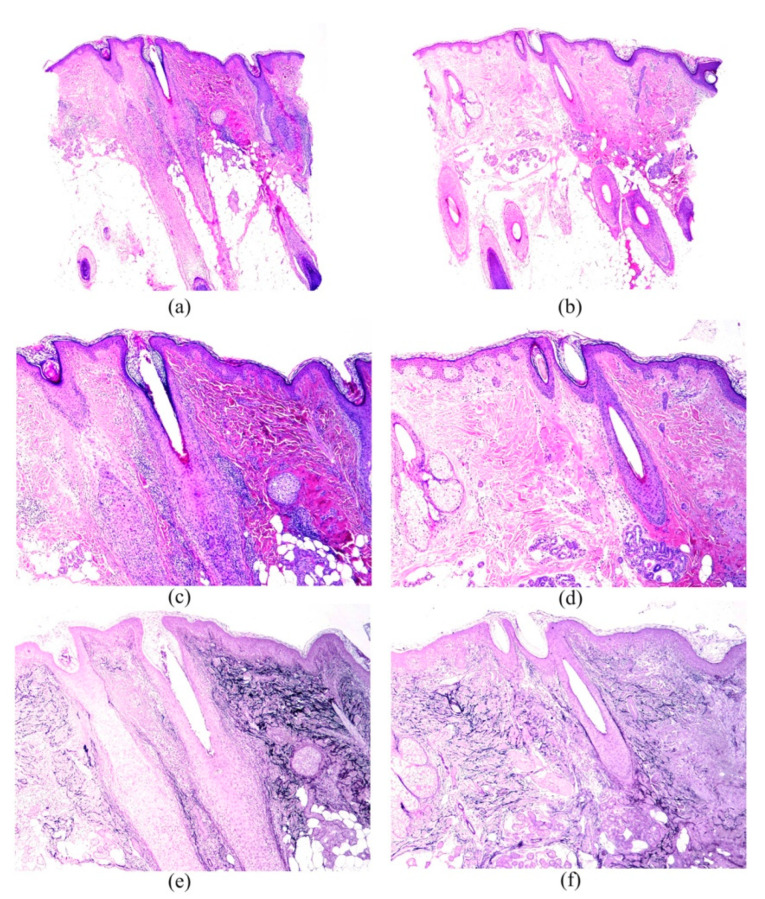
Images from patient number 47. (**a**,**c**,**e**) show the biopsy from the most clinically involved area (frontal hairline). Although lymphoid infiltrate is not as prominent as in case 50, scarring areas are extensive in the peri-infundibular zone. ((**a**) Hematoxylin-eosin × 20; (**c**) Hematoxylin-eosin × 40; (**e**) Shikatta orcein × 40). (**b**,**d**,**f**) show the biopsies from a normal-appearing scalp area. The scarring areas are much less prominent, and so is the solar elastosis. ((**b**) Hematoxylin-eosin × 20; (**d**) Hematoxylin-eosin × 40; (**f**) Shikatta orcein × 40).

The characteristics of the inflammatory infiltrate were presented in Table 3. All types of hair follicles—terminal, intermediate, and vellus—were affected by the inflammatory infiltrate in both areas (Figure 1a–d and Figure 2a–d), although terminal hair follicles were the most commonly involved (71.15%—37/52 in B1 vs. 36.54%—19/52 in B2). Hair follicles were not affected by inflammatory infiltrate in 25% (13/52) of B1 samples compared to 46.2% (24/52) in B2 (*p* = 0.027). The inflammatory infiltrate also involved hair follicles in all phases of the hair cycle in both areas, although as anagen hairs were the most frequently present, they were also the most frequently infiltrated (55.8%—29/52 in B1 vs. 51.9%—27/52 in B2). Dermal involvement included, in most cases, papillary and reticular dermis (61.5%—32/52 in B1 and 38.5%—20/52 in B2) (Table 3). The inflammatory infiltrate also involved the interfollicular space, mainly with a perifollicular and perivascular distribution. A lichenoid infiltrate in the interfollicular epidermis was only found in one patient in B1.

Epithelial changes are shown in Table 4. Corneum stratum changes were noted as hyperkeratosis or follicular plugs, whereas epidermal changes were observed as hyperplasia in both areas, and also vacuolar changes and atrophy in B1. Follicular epithelium changes (Figure 1a,c) were more commonly seen than the other epithelial changes, especially in B1, and the most frequent type, in both areas, was the vacuolar change (62.7%—32/51 in B1 and 40.4%—21/52 in B2), followed, at a considerable distance, by lichenoid changes, cysts, spongiosis, and grouping of follicles. Colloid bodies in the dermoepidermal junction were found in only 5.9% (3/51) of the B1 samples.

Fibrous tissular changes are also presented in Table 4. Terminal, intermediate, and vellus hairs were affected by perifollicular fibrosis, although the former type was the most frequently involved (65.4%—34/52 in B1, and 21.2%—11/52 in B2). Dermal fibrosis was located, most commonly, in the upper dermis, and was generally noted as follicular lamellar concentric fibrosis and fibroplasia. Fibrous tracts were noted in both areas, and no significant differences were found between them. Interfollicular mucinosis was noted in only one patient in B2, whereas mild follicular mucinosis was observed in 9.6% (5/52) and 7.7% (4/52) of B1 and B2 samples, respectively. Elastic fibers pattern was generally normal (69.2%—36/52 in B1 and 67.3%—32/52 in B2), although in some they were destructed (Figure 1e,f and Figure 2e,f). Foreign reaction granulomas were noted in some patients, in both B1 and B2.

Patients with follicular hyperkeratosis and perifollicular erythema more frequently had inflammatory infiltrate in both areas, although the difference did not reach statistical significance. No association was noted between the presence of histological inflammation and the presence of symptoms. Involvement of other skin annexes rather than hair follicles (sebaceous glands and erector pili muscle) in B1 were observed in 63.6% (7/11) of patients with facial papules compared to 92.7% (38/41) of patients without facial papules (*p* = 0.029). Vacuolar degeneration of the basal layer of the outer root sheath in B2 was seen in 53.8% (14/26) of the patients with an earlier debut of the alopecia compared to 19.2% (5/26) of patients with a later debut (*p* = 0.020). Fibrous tissular changes in B1 were more frequent in patients with absence of vellus hair in the frontal hairline (84%—42/50—), compared to those with presence of vellus hairs in which fibrous changes were not observed (*p* = 0.034). Regarding the use of corticosteroids, no differences were noted in the presence of the inflammatory infiltrate in B1 or in B2, in patients who were undergoing maintenance therapy, compared to those not using corticosteroids.

## 4. Discussion

All the histopathological features described in FFA were observed in both the frontal active hairline and the “normal-appearing” scalp, although some of them were less frequent or to a lesser degree in the latter. The largest histopathological study so far regarding features in normal-appearing scalp in FFA included 28 patients, and also studied vertical sections of the biopsies [14].

A lower number of hair follicles would be expected as a consequence of the scarring process: Chew et al. found that a mean of seven terminal hair follicles were observed per 4 mm punch biopsy in FFA patients, while around thirty terminal and five vellus hair follicles would be a common finding in healthy Caucasians [15]. The mean follicular number found in the frontal hairline in our patients was lower, which may be due to a more severe alopecia among them. Interestingly, the mean follicular count found in the “healthy scalp” was also reduced compared to a truly healthy scalp, although the older age of our patients could be partly responsible for that [16]. A significant reduction in total follicular count and in terminal follicular count was observed in the frontal hairline compared to the “healthy scalp”.

The loss of sebaceous glands is an early finding in FFA, and their atrophy along with the inflammatory involvement of the vellus follicles may be the histologic clues in these early phases [17]. Atrophic sebaceous glands have also been found in normal-appearing scalp in FFA [11]. In the present report, sebaceous gland reduction or absence were noted in both areas, but this impairment was significantly more common in the frontal hairline. The erector pili muscle was less often affected.

Perifollicular lymphocytic inflammation around the isthmus/infundibulum in “healthy scalp” was found, in accordance with previous reports [11,12,14]. Around 86% of patients showed this feature, a higher percentage than that reported by Doche et al. (64.3%) [14]. As stated by previous studies [13], which also used vertical sections, no significant differences were found between the presence of the inflammatory infiltrate in the “healthy scalp” compared to the frontal hairline area, although there were significant differences regarding the severity of the infiltrate, which was milder in the former.

All types of follicles in all phases of the hair cycle were affected in the same way by the inflammatory infiltrate in both areas, in accordance with previous reports [7], although terminal and anagen hairs were the most commonly affected.

Unlike previous reports, which found that the interfollicular epidermis was usually spared by the inflammatory infiltrate [7,8], around 86% and 71% of the patients in the current study had interfollicular involvement in the frontal hairline and the “healthy scalp”, respectively, and it was mainly with a perifollicular and perivascular distribution. Vacuolar degeneration of the basal layer is another FFA sign [7]. In our patients, follicular epithelium changes were significantly more frequent in the frontal hairline and the main type was the vacuolar degeneration. Keratinocyte necrosis in the external root sheath is also a prominent feature in FFA, especially at the isthmus [18]. This finding was significantly more frequent in the frontal hairline, where an increased apoptotic activity in the outer root sheath was also seen more often.

Doche et al. found perifollicular fibrosis in normal-appearing areas in FFA in almost 18% of patients [14]. In the present study, fibrous tissular changes were commonly seen in both areas, but significantly more often in the frontal hairline. Loss of elastin fibers has also been described as a finding associated with the fibrosis [8], although in our patients, the elastic fiber pattern was generally normal. Doche et al. found mucin deposits in the affected hairline in 35.7% of patients and in 7.1% in the “healthy scalp” [14], a higher frequency than that found in the current study, in the frontal hairline, and a similar frequency, in the “healthy scalp”.

The presence of inflammatory infiltrate in both areas tended to be more frequent in patients with trichoscopic inflammatory signs, but it did not reach statistical significance. This could be explained by the low number of patients without trichoscopic inflammatory signs or because the association between both may not be real. Involvement of sebaceous glands/erector pili muscle in the frontal hairline was significantly less prevalent in patients with facial papules. One explanation could be that facial papules may appear early in the course of the disease, as was proposed by some authors [19], maybe even earlier than the alteration of these skin annexes appears. Moreover, patients who maintained vellus hairs in the frontal hairline had no fibrous tissular changes in this area, which is compatible with the clinical observation that vellus hair may be clinically present in incipient FFA cases [20].

Although the involvement of “normal-appearing scalp” and the presence of peripheral alopecia may suggest that FFA is a generalized inflammatory process, a recent study has found that FFA has scalp immunity and fibrosis dysregulation but without systemic involvement [21]. The reason why this normal-appearing scalp, with histopathological evidence of the disease, does not develop alopecia like the frontal hairline does, is still unknown; the frontal hairline may be more susceptible to the hair loss than the parietal area.

The limitations of this study included the cross-sectional design, without a follow up of the histopathological changes, and the fact that some patients were receiving treatment with topical corticosteroids. However, they were using them as a low-frequency maintenance therapy, and no differences were found regarding the presence of inflammatory infiltrate in patients who were using corticosteroids compared to the non-users. Another limitation could be that the scalp biopsies were only assessed on vertical sections.

In conclusion, all of the histopathological features of FFA were also present in the supposedly healthy scalp. Further studies are needed to clarify why these areas do not develop alopecia despite having the same histopathological features as the affected hairline.

## Figures and Tables

**Table 1:** Clinical and demographic characteristics of FFA patients.

n: 52	Mean
Age (years)	63.04 (SD 9.69)
Age of menopause (years)	50.5 (SD 3.61)
Age of onset of the alopecia (years)	58.42 (SD 10.17)
Duration of the alopecia (months)	55.58 (SD 37.63)
	**Frequencies**
Menopause	88.5% (46/52)
Pruritus	71.2% (37/52)
Trichodynia	23.1% (12/52)
Occipital involvement	9.6% (5/52)
Eyebrow alopecia	86.5% (45/52)
Facial papules	21.2% (11/52)
FFA severity grade:	
Grade I	5.8% (3/52)
Grade II	38.5% (20/52)
Grade III	36.5% (19/52)
Grade IV	11.5% (6/52)
Grade V	7.7% (4/52)
Perifollicular erythema	84.6% (44/52)
Follicular hyperkeratosis	88.5% (46/52)

**Table 2:** Follicular count in both areas, frontal hairline (B1) and healthy scalp (B2), on vertical sections.

N52	Mean (SD)		
Follicular Count	Frontal Hairline (B1)	Healthy Scalp (B2)	*p* Value
Total	5.10 (2.77)	7.79 (3.87)	<0.001
Terminal hairs	4.25 (2.59)	6.58 (3.48)	<0.001
Intermediate hairs	0.38 (0.91)	0.50 (1.16)	0.479
Vellus hairs	0.46 (0.85)	0.71 (0.98)	0.166
Anagen hairs	4.67 (2.90)	7.35 (3.80)	<0.001
Telogen hairs	0.44 (0.67)	0.44 (0.78)	1.000

**Table 3:** Presence of inflammatory infiltrate and its characteristics in both areas.

N52	Frontal Hairline (B1)	Healthy Scalp (B2)	*p* Value
Inflammatory infiltrate (yes)	92.3% (48/52)	86.5% (45/52)	0.508
Severity			
Mild	35.4% (17/48)	71.1% (32/45)	0.013
Moderate	60.4% (29/48)	26.7% (12/45)	
Severe	4.2% (2/48)	2.2% (1/45)	
**Specific location of the inflammatory infiltrate**			
Sebaceous gland (yes)	13.5% (7/52)	5.8% (3/52)	0.368
Dermis (general) (yes)	88.5% (46/52)	71.2% (37/52)	0.035
Interfollicular dermis (yes)	86.5% (45/52)	71.2% (37/52)	0.057
Superficial perivascular lymphohistiocytic infiltrate	86.4% (44/52)	69.2% (36/52)	0.039

The evaluation of the infiltrate was first made in general terms and at low power (mild/moderate/severe) and it was then specified the location of the infiltrate.

**Table 4:** Epithelial changes and fibrous tissular changes.

	Frontal Hairline (B1)	Healthy Scalp (B2)	*p* Value
**Epithelial changes**			
Corneum stratum changes (including hyperkeratosis, parakeratosis or both) (yes)	11.5% (6/52)	24.5% (12/49)	0.180
Epidermal changes (specified below) (yes)	17.3% (9/52)	6.1% (3/49)	0.07
Follicular epithelium changes (specified below) (yes)	70.6% (36/51)	48.1% (25/52)	0.012
Interface dermatitis lichenoid in the upper part of the hair follicle	23.5% (12/51)	15.4% (8/52)	0.125
Vacuolar degeneration basal layer outer root sheath	64.7% (33/51)	36.5% (19/52)	0.001
Necrosis of keratinocytes outer root sheath	43.1% (22/51)	15.4% (8/52)	0.001
Increased apoptotic activity in outer root sheath (more intense than the physiological grade commonly seen in any biopsy)	45.1% (23/51)	19.2% (10/52)	0.001
Infundibular dilatation and infundibular hypergranulosis	13.7% (7/51)	7.7% (4/52)	0.508
**Fibrous tissular changes**	80.8% (42/52)	53.8% (28/52)	0.003
Perifollicular fibrosis	71.2% (37/52)	30.8% (16/52)	<0.001
Interfollicular dermal fibrosis	53.8% (28/52)	36.5% (19/52)	0.122

Corneum stratum changes such as hyperkeratosis or follicular plugs, epidermal changes such as hyperplasia, vacuolar changes or atrophy. Follicular epithelium changes such as vacuolar changes, lichenoid changes, cysts, spongiosis, or tufted hairs.

## Data Availability

Not applicable.
